# Using record linkage to monitor equity and variation in screening programmes

**DOI:** 10.1186/1471-2288-12-59

**Published:** 2012-04-25

**Authors:** Dermot O’Reilly, Heather Kinnear, Michael Rosato, Adrian Mairs, Clare Hall

**Affiliations:** 1 Centre for Public Health, Queen’s University Belfast, Belfast, Northern Ireland; 2 Public Health Agency, Belfast, Northern Ireland; 3 Centre for Public Health, Queen’s University Belfast, Mulhouse Building, Grosvenor Road, Belfast, BT12 6BJ, Northern Ireland

**Keywords:** Data linkage, Breast screening, Inequalities, Equity monitoring

## Abstract

**Background:**

Ecological or survey based methods to investigate screening uptake rates are fraught with many limitations which can be circumvented by record linkage between Census and health services datasets using variations in breast screening attendance as an exemplar. The aim of this current study is to identify the demographic, socio-economic factors associated with uptake of breast screening.

**Methods:**

Record linkage study: combining 2001 Census data within the Northern Ireland Longitudinal Study (NILS) with data relating to validated breast screening histories from the National Breast Screening System. A cohort was identified of 37,059 women aged 48-64 at the Census who were invited for routine breast screening in the three years following the Census. All cohort attributes were as recorded on the Census form.

**Results:**

The record linkage methodology enabled the records of almost 40,000 of those invited for screening to be analysed at an individual level, exceeding the largest published survey by a factor of ten. This produced a more robust analysis and demonstrated (in fully adjusted models) the lower uptake amongst non-married women and those in the lowest social class (OR 0.74; 95%CI 0.66, 0.82), factors that had not been reported earlier in the UK. In addition, with the availability of both individual and area information it was possible to show that the much lower screening uptake in urban areas is not due to differences in population composition suggesting unrecognised organisational problems.

**Conclusions:**

Linkage of screening data to Census-based longitudinal studies is an efficient and powerful way to increase the evidence base on sources of variation in screening uptake within the UK.

## Background

Cancer survival rates in the UK are not as good as in other parts of Europe and there is a body of evidence showing that this is, to some extent, because patients here tend to present at diagnosis with more advanced disease than their European peers [1,2]. One of the best ways to increase the rates of early cancer detection is through screening [3] and in the UK there are national population screening programmes for breast, cervical and more recently colorectal cancer. Although the effectiveness of these screening programmes depends on rates of participation, routine information on factors affecting uptake rates is limited. A systematic review of studies on inequalities in access to cancer services published between 1998 and 2003 [4] concluded that “there is a dearth of information related to variations in uptake in UK”.

The few UK studies examining variations in screening uptake and coverage have had significant methodological limitations either because they used ecological measures of socio-economic status [5,6] or have been based on surveys [7,8]. For example, in the largest UK study published to date, Moser et al [9] examined reported use of breast and cervical screening in 3,185 women interviewed as part of the National Statistics Omnibus Surveys (2005-07). They found that car availability and housing tenure were associated with uptake of breast screening but not with cervical screening (though the effect sizes were suggestive). However, with response rates of 64-69%, there are concerns surrounding representativeness. In addition, the reliance on self-reported information introduces the possibility of recall bias and reporting error, and even after combining nine surveys the relatively small study size limited the extent of the analyses so that the recognized lower screening uptake in larger conurbations such as London [10,11] could not be confirmed. Similar difficulties are apparent with research relating to screening for colorectal cancer; in some studies just over half of those eligible for screening responded [12], and others have had to use ecological proxies for either deprivation [13] or ethnicity [14].

The deficiencies in the current methods for assessing and monitoring screening programmes and the need for further high quality information to inform the screening programmes has been highlighted by the UK National Screening Programmes Information Strategy [15] which concluded … “Assessing equity is a particular concern as analyses by variables currently available fall short of the ideal”, or as Weller and Campbell [16] have stated “…effective and efficient cancer screening programmes based on sound evidence are needed.”

The aim of the current study is to show how many of the difficulties associated with the current ecological or survey based methods can be circumvented by record linkage between Census and health services datasets using variations in breast screening attendance as an exemplar.

## Methods

Information about eligibility and uptake of breast screening in Northern Ireland is held by the Quality Assurance Reference Centre (QARC) which is responsible for coordinating breast screening in Northern Ireland for the UK-wide National Breast Screening System. This information includes: date of birth; address; date of invitation; date of attendance; whether the attendance was based on routine recall, GP referral or self referral; and information relating to screening results and, where necessary, referrals for further consultations or treatment. The dataset also contains the Health and Care Number (the unique NHS identifier) which is important for linkage (see below). The linkage methodology was adapted from the one Scotland had previously used when linking their hospital and morbidity records to their Census returns [17]. At the time of the study only women aged 50-64 were routinely invited for breast screening so the cohort for analysis was defined as all women aged 48 to 64 years at the time of the 2001 Census who had been invited for routine screening (including GP and self referrals) in the three years following the Census (April 2001-March 2004). This excluded early rescreens, technical repeats and women who had been referred from initial screening for investigations and/or treatment. Uptake rates were calculated as the total number of women who attended during this period (plus a 6 month slippage time to allow for delayed attendance), divided by the total number of women invited during the three year screening cycle.

The Northern Ireland Longitudinal Study (NILS) is a representative sample of approximately 28% of the population (nearly 500,000 people) formed from the linkage of the Health Card registration system and the 2001 Census returns [18]. NILS is maintained by the Northern Ireland Statistics and Research Agency (NISRA). It is modeled on the Office for National Statistics Longitudinal Study (ONS-LS) [19] and is similar to the Scottish Longitudinal Study [20]. The core NILS database includes cohort members’ Census records and contextual information relating to household composition and the characteristics of the area of residence. It also contains the Health and Care Number that enables unique linkage to other health service databases including the National Breast Screening System. The screening data and NILS were linked using the encrypted Health and Care number as the matching field, a process carried out jointly by the respective data custodians within the secure setting in NISRA, after which the matching field, and other identifiers, were removed. The dataset was then anonymised before being supplied to the researchers, and was held in a secure setting by the Registrar General. At no time were patient identifiable data available. The approval for the record linkage by the data custodians in the respective organisations was facilitated by the obvious policy relevance of the study and underpinned by detailed data transfer agreements. The study was approved by the Office for Research Ethics Committee (ORECNI), the local ethics committee in Northern Ireland (ref: 07/NIR01/90).

All characteristics of women in the cohort were as recorded on the Census form and selected as factors shown from other studies to be associated with screening uptake. Age was included as 5-year bands (≤49, 50-54, 55-59, 60-64); marital status was categorised as married/co-habiting, never married, and a final group combining the widowed, separated or divorced (as analyses showed similar levels of uptake in these women). Household composition (dichotomised as single person household or not) was also included. Four census-based indicators of socio-economic status were included: the National Statistics Socio-Economic Classification (six bands see Table 1) [21]; highest educational attainment (ranked as: university level, ‘A-level’, ‘O-level’ or their equivalents, and no qualifications); household car availability (two or more cars, one car, no car access) and housing tenure (owner occupier, private renter and social renter). Two census-based measures of self reported morbidity were also included; one on the presence of limiting long term illness (with a yes/no response) and another on general health in the year preceding the Census which offered three responses – good, fairly good and not good. An indicator of the urban/rural character of the area in which the respondent lived was included, divided into three bands; urban (population size greater than 75,000), intermediate (population size between 2250 and 75,000) and rural (population size less than 2250) [22].

**Table 1:** Demographic and socio-economic factors associated with attendance at breast screening

		**No Invited (%Pop)**	**No Attended (%Uptake)**
**Age at census**	**≤ 49**	4,249 (11.5)	2,978 (70.1)
	**50-54**	12,902 (34.8)	10,075 (78.1)
	**55-59**	11,888 (32.1)	9,084 (76.4)
	**60-64**	8,020 (21.6)	5,684 (70.9)
**Marital Status**	**Married**	26,967 (72.8)	21,029 (78.0)
	**Never married**	2,774 ( 7.5)	1,851 (66.7)
	**Sep/Wid/Div**	7,318 (19.8)	4,941 (67.5)
**National Statistics Socio-economic Classification**	**H/L Prof/Manual**	9,198 (24.8)	7,124 (77.5)
	**Intermediate**	5,555 (15.0)	4,297 (77.4)
	**Own Account**	1,958 ( 5.3)	1,527 (78.0)
	**Lower Supervisory**	2,058 ( 5.6)	1,557 (75.7)
	**Routine**	15,775 (42.6)	11,708 (74.2)
	**Other-U/E**	2,515 ( 6.8)	1,608 (63.9)
**Car Access**	**2 and over**	16,116 (43.5)	12,948 (80.3)
	**1 car**	15,442 (41.7)	11,567 (74.9)
	**No car**	5,501 (14.8)	3,306 (60.1)
**Housing tenure**	**Owner**	30,044 (81.1)	23,446 (78.0)
	**Private rent**	1,340 ( 3.6)	897 (66.9)
	**Social rent**	5,675 (15.3)	3,478 (61.3)
**Education**	**Degree+**	4,138 (11.2)	3,203 (77.4)
	**To A-Level**	1,411 ( 3.8)	1,068 (75.7)
	**To GCSE**	8,676 (23.4)	6,791 (78.3)
	**None**	22,834 (61.6)	16,759 (73.4)
**General Health**	**Good**	19,101 (51.5)	14,826 (77.6)
	**Fairly good**	10,616 (28.7)	7,994 (75.3)
	**Not good**	7,342 (19.8)	5,001 (68.1)
**Limiting long term illness**	**No**	25,245 (68.1)	19,471 (77.1)
	**Yes**	11,814 (31.9)	8,350 (70.7)
**Residence**	**Urban**	14,640 (39.5)	10,107 (69.0)
	**Intermediate**	12,399 (33.5)	9,666 (78.0)
	**Rural**	10,020 (27.0)	8,048 (80.3)

*Fully adjusted for age, marital status, NSSEC, car access, housing tenure, educational attainment, general health, limiting long-term illness and settlement band.

Multivariate logistic regression using STATA version 10 was used to explore the relationship between uptake of breast screening and the socio-demographic and socio-economic variables (Table 2). The odds ratios (and 95% confidence intervals) for each of the regression models are presented in this paper.

**Table 2:** Demographic and socio-economic predictors of attendance at breast screening. Numbers in bold indicate odds ratios that were significant at the p < 0.05 level

		Model 1*	Model 2*	Model 3*	Model 4*	Model 5*
**Age at census**	**≤ 49**	1.00	1.00	1.00	1.00	1.00
	**50-54**	**1.52 (1.41-1.64)**	**1.51 (1.40-1.63)**	**1.55 (1.43-1.67)**	**1.56 (1.44-1.69)**	**1.57 (1.45-1.70)**
	**55-59**	**1.38 (1.28-1.50)**	**1.38 (1.27-1.49)**	**1.43 (1.32-1.55)**	**1.46 (1.34-1.58)**	**1.47 (1.36-1.60)**
	**60-64**	1.04 (0.96-1.13)	1.06 (0.97-1.15)	**1.12 (1.03-1.21)**	**1.13 (1.03-1.22)**	**1.13 (1.04-1.23)**
**Marital Status**	**Married**	1.00	1.00	1.00	1.00	1.00
	**Never married**	**0.57 (0.52-0.62)**	**0.57 (0.52-0.62)**	**0.75 (0.69-0.82)**	**0.75 (0.68-0.82)**	**0.74 (0.67-0.81)**
	**Sep/Wid/Div**	**0.60 (0.56-0.63)**	**0.60 (0.56-0.63)**	**0.84 (0.79-0.90)**	**0.84 (0.79-0.90)**	**0.84 (0.79-0.90)**
**National Statistics Socio-economic Classification**	**H/L Prof/Manual**	1.00		1.00	1.00	1.00
	**Intermediate**	1.00 (0.92-1.10)		1.01 (0.93-1.10)	1.00 (0.93-1.10)	1.03 (0.94-1.12)
	**Own Account**	1.03 (0.91-1.16)		0.98 (0.87-1.11)	0.98 (0.87-1.11)	0.93 (0.82-1.05)
	**Lower Supervisory**	0.90 (0.81-1.01)		1.07 (0.95-1.21)	1.09 (0.96-1.22)	1.07 (0.95-1.20)
	**Routine**	**0.84 (0.79-0.89)**		1.03 (0.96-1.11)	1.04 (0.96-1.12)	1.02 (0.94-1.10)
	**Other-U/E**	**0.52 (0.47-0.57)**		**0.77 (0.69-0.86)**	**0.78 (0.70-0.87)**	**0.74 (0.66-0.82)**
**Car Access**	**2 and over**	1.00		1.00	1.00	1.00
	**1 car**	**0.74 (0.70-0.78)**		**0.84 (0.79-0.89)**	**0.85 (0.80-0.90)**	**0.89 (0.84-0.95)**
	**No car**	**0.37 (0.35-0.39)**		**0.56 (0.51-0.61)**	**0.58 (0.53-0.63)**	**0.63 (0.58-0.69)**
**Housing tenure**	**Owner**	1.00		1.00	1.00	1.00
	**Private rent**	**0.57 (0.51-0.65)**		**0.71 (0.63-0.80)**	**0.72 (0.64-0.81)**	**0.68 (0.61-0.77)**
	**Social rent**	**0.44 (0.42-0.47)**		**0.64 (0.60-0.69)**	**0.66 (0.62-0.71)**	**0.67 (0.63-0.73)**
**Education**	**Degree+**	1.00		1.00	1.00	1.00
	**To A-Level**	0.91 (0.79-1.05)		0.92 (0.79-1.06)	0.92 (0.80-1.07)	0.92 (0.79-1.06)
	**To GCSE**	1.05 (0.96-1.15)		1.08 (0.98-1.19)	0.99 (0.97-1.19)	1.06 (0.97-1.17)
	**None**	**0.80 (0.74-0.87)**		1.00 (0.91-1.10)	1.02 (0.92-1.12)	0.99 (0.90-1.09)
**General Health**	**Good**	1.00			1.00	1.00
	**Fairly good**	**0.89 (0.84-0.94)**			1.00 (0.94-1.06)	1.00 (0.94-1.06)
	**Not good**	**0.61 (0.58-0.65)**			**0.79 (0.72-0.86)**	**0.81 (0.74-0.89)**
**Limiting long term illness**	**No**	1.00			1.00	1.00
	**Yes**	**0.72 (0.68-0.75)**			1.01 (0.94-1.09)	1.01 (0.94-1.08)
**Residence**	**Urban**	1.00				1.00
	**Intermediate**	**1.59 (1.51-1.68)**				**1.56 (1.47-1.65)**
	**Rural**	**1.83 (1.72-1.94)**				**1.54 (1.45-1.64)**

*Model 1 – age only adjustment. Model 2 – adjustment for age and marital status. Model 3 – adjustment for age, marital status and socio-economic status. Model 4 – adjustment for age, marital status, socio-economic status and health status. Model 5 – adjustment for age, marital status, socio-economic status, health status and area of residence.

## Results

The linked dataset included a total of 37,059 women who, during the three year period (2001-2004), had been invited for breast screening; 11,931 aged 48-52 at the time of the 2001 Census invited for their first screen and 25,128 women aged 53-64 at the time of the 2001 Census who had been invited for routine subsequent screens during the study period. 11.5% of the population were aged less than 50 at the time of the 2001 Census but would have received an invitation to attend their first breast screening appointment during the time period of the study when they reached 50 years of age. The routine screening uptake rate for women included in the Census based cohort during this three year cycle was 75.1%, comparing favourably with the QARC estimate of 74.6% for all women screened in Northern Ireland during the same period. Uptake for women aged 48-52 who had their first invitation during this time was 75.2%.

The factors associated with lower uptake for all women invited for screening also applied to first-time invitees. Consequently, only the former are described in detail. Table 1 shows that uptake rates were highest in the 50-59 year olds and about 8% lower for women aged either less than 50 or aged 60 and over. Married women constituted approximately 70% of this age group and these women had higher uptake rates than either those who were never married (OR = 0.74; 95% CI = 0.67, 0.81) or those who were widowed, separated or divorced (OR = 0.84; 95% CI = 0.79, 0.90).

Uptake of screening was strongly linked to both car availability and housing tenure in the fully adjusted model, but not to educational attainment or occupational social class (NSSEC). Compared to owner occupiers, social renters (who comprised 15.3% of the cohort) were about one third less likely to have attended for screening (OR = 0.67; 95% CI = 0.63, 0.73) with little difference between social and private renters. Compared to women with household access to more than two cars: those with access to one car only (41.7% of the cohort) were less likely to attend screening (OR = 0.89; 95% CI = 0.84, 0.95); while those with no car access (14.8% of the cohort) were least likely to attend (OR = 0.63; 95% CI = 0.58, 0.69). There was no significant interaction between car access and urban/rural residence (chi-square = 2.67, p = 0.615) indicating that the association between screening attendance and car access was similar in urban and rural areas. There was no association between educational attainment and invitation for screening - in the fully adjusted model women with no formal qualifications (62% of the cohort) were as likely as those with a degree (12% of cohort) to have attended. No gradients in uptake were apparent across social class, though those classified as ‘Other’ (comprising 6% of the cohort -including those classified as having ‘never worked’ and the long-term unemployed) were less likely than those in the professional & managerial occupations to have attended for screening (OR = 0.74; 95% CI = 0.66, 0.82).

Women with poorer health at the time of the census were less likely to attend for screening within the next three years; eg OR = 0.72 (0.68-0.75) for those reporting a limiting long-term illness, after adjustment for age. However, when both indicators of self-reported morbidity were included in the fully adjusted model only general health remained significantly associated with screening uptake: those reporting’not good’ health were less likely to attend than those reporting good health (OR = 0.81; 95% CI = 0.74, 0.89).

## Discussion

To our knowledge this is the first time that validated screening records have been linked to Census returns and the results have produced the largest and most representative individual-level study of factors associated with uptake of breast screening in the UK to date. The overall attendance rate in the period covered by the study (2001-04) was 75.1%, lower than the national screening target of 80%, but higher than the national average for 2008-09 which was 73.9% [10]. However, attendance rates have increased since then and at 76.4% (for 2008/09), Northern Ireland now compares favourably with the rest of the UK [23,24].

The record linkage methodology enabled the records of almost 40,000 of those invited for screening to be analysed at an individual level, exceeding the largest published survey by a factor of ten. The findings both confirm and add to what was previously known about the social and socio-economic factors influencing screening attendance. The lower uptake at older ages persists after adjustment for other factors such as health status and is worrying given the increased incidence of cancer amongst older people. Uptake in the 65-69 year range was not measured as the extension of breast screening to older ages had not yet been introduced in Northern Ireland. The higher breast screening uptake amongst women who are currently married has been found in other countries [25], but to our knowledge this is the first study to report it in the UK.

While the study reaffirms the relationship between attendance for breast screening and socio-economic status, it also suggests that this is not due to potential confounders such as health status. Not all indicators of disadvantage were important: uptake was related to car availability and housing tenure but not to educational attainment or occupational social class (with the exception of those who never worked and the long-term unemployed). Interpretation of indicators of disadvantage is difficult and explanations other than those related to deprivation are possible [26]: while the association with car availability might suggest difficulties with accessing screening facilities, the availability of both individual and area factors enabled us to show that as there is a similar relationship between screening uptake and car ownership in both rural and urban areas (the latter with good public transport networks), that socio-economic factors, rather than the simpler issue of transport, may be more important. Similarly, housing tenure represents more than wealth and encompasses aspects of the physical and social environment of residence that can shape lifestyle choices and health behaviours [27]. The absence of any relationship between screening uptake and educational attainment confirms findings of other UK-based studies [9,23]. This might imply either that prior attainment (maybe thirty years previously) is not closely related to current socio-economic status, or that the socio-economic gradients are related to health beliefs and attitudes rather than a lack of knowledge.

The health of the women at baseline was also important in determining attendance at screening though, surprisingly, the relationship was with general health rather than limiting long-term illness (LLTI). Why this is so is unknown, though it is possible that the more general self-rated health measure can more easily capture a contribution to future ill-health if the perception of poorer health leads to less engagement with preventative practices (such as attendance at screening) [28].

### Strengths and Limitations

The major strengths of this study is the size and quality of the linked datasets and because the linkage is of routine administrative databases there is no responder burden so responder bias is not a problem. The National Breast Screening System provides validated uptake rates and makes it possible to follow women through different screening cycles differentiating between occasional and recalcitrant non-attenders. The legal obligation to complete the Census ensures that it the largest and most representative description of the population and the availability of measures of socio-economic status at individual or household level have circumvented many limitations of earlier studies that have relied on surveys with moderate responses or ecological measures of socio-economic status. This larger size has made it possible to include a greater range of factors that could have potentially confounded the results of earlier studies. One of the major strengths of this study was the ability to incorporate both individual and area factors to demonstrate that the markedly lower uptake in urban areas is not due to differing population compositions. This could not have been ascertained using either surveys or ecological methods, and hints at organizational difficulties around cities. The Census also complements the screening data for factors such as ethnicity that are not well captured by the NHS. (This was not examined in the current study as the prevalence of ethnic minorities in Northern Ireland was <1%).

There are some limitations to the record linkage approach. The exploration of factors likely to influence screening uptake is dependent on the Census variables though these are generally more extensive than in most datasets, with the exception of health surveys. A further caveat is that although the Census is the largest and most representative survey of the population, it does not include everyone. It is estimated that about 5% of the UK population were not enumerated at the last Census, though this is primarily a problem amongst younger adults, males, and residents in more deprived inner city areas. Arguably the utility of a link to the Census wanes with distance from Census as cohort characteristics may change. This may be a particular problem for some factors such as health and marital status but less so for socio-economic status, which for the age-groups being screened for chronic disease is reasonably stable. In any event, detailed monitoring may only be needed episodically.

### Conclusions

This study demonstrates that linkage of data from screening programs to Census data can provide a powerful and efficient means of monitoring inequalities in uptake of screening. Although the study was conducted in Northern Ireland, both the approach and the general findings have relevance to the rest of the UK. The linkage of these large and sensitive datasets involves some effort for researchers but the presence of large Census-based longitudinal studies in both Scotland and England and Wales should facilitate the process, opening up the potential for regular and detailed monitoring of screening for breast, cervical and colorectal cancer as well as for other non-cancer screening programs. The methodology could also be extended to study socio-economic and area-level factors influencing other aspects of health service utilization.

## Competing interests

The authors declare that they have no competing interests.

## Authors’ contributions

HK and DOR designed the study. HK with the assistance of MR undertook all of the analysis. All authors contributed to the interpretation of the results and the writing of the paper. All authors had full access to all of the data during the course of the study and can be held responsible for the integrity and accuracy of the data. All authors read and approved the final manuscript.

## Pre-publication history

The pre-publication history for this paper can be accessed here:

http://www.biomedcentral.com/1471-2288/12/59/prepub
